# Micelle Structure Details and Stabilities of Cyclic Block Copolymer Amphiphile and Its Linear Analogues

**DOI:** 10.3390/polym11010163

**Published:** 2019-01-17

**Authors:** Brian J. Ree, Toshifumi Satoh, Takuya Yamamoto

**Affiliations:** 1 Graduate School of Chemical Sciences and Engineering, Hokkaido University, Sapporo 060-8628, Japan; brianree@poly-bm.eng.hokudai.ac.jp; 2 Faculty of Engineering, Hokkaido University, Sapporo 060-8628, Japan

**Keywords:** synchrotron X-ray scattering, cyclic block copolymer, linear triblock copolymer, linear diblock copolymer, amphiphiles, micellar morphology, structural parameters, micelle stability, topological effect

## Abstract

In this study, we investigate structures and stabilities of the micelles of a cyclic amphiphile (*c*-PBA-*b*-PEO) composed of poly(*n*-butyl acrylate) (PBA) and poly(ethylene oxide) (PEO) blocks and its linear diblock and triblock analogues (*l*-PBA-*b*-PEO and *l*-PBA-*b*-PEO-*b*-PBA) by using synchrotron X-ray scattering and quantitative data analysis. The comprehensive scattering analysis gives details and insights to the micellar architecture through structural parameters. Furthermore, this analysis provides direct clues for structural stabilities in micelles, which can be used as a good guideline to design highly stable micelles. Interestingly, in water, all topological polymers are found to form ellipsoidal micelles rather than spherical micelles; more interestingly, the cyclic polymer and its linear triblock analog make oblate-ellipsoidal micelles while the linear diblock analog makes a prolate-ellipsoidal micelle. The analysis results collectively inform that the cyclic topology enables more compact micelle formation as well as provides a positive impact on the micellar structural integrity.

## 1. Introduction

In polymer science, synthetic methodologies have advanced significantly over the last two decades [1,2,3,4,5,6,7,8,9,10,11,12,13,14,15,16,17]. As a result, there are several synthetic strategies for producing cyclic topological polymers [6,7,8,9,10,11,12,13,14,15,16,17]. Nevertheless, these synthetic methods are still facing key challenges such as the minimization of side reactions and the resulting byproducts, and improvement of workup processes for providing highly pure cyclic polymers while retaining high yields. With regard to the characterization perspective, many physicochemical investigations of synthesized cyclic polymers have been proceeded in the aspect of cyclic topological effects on the chain and morphological structures and properties [17,18,19,20,21,22,23,24,25,26,27,28]. In particular, amphiphilic cyclic block copolymers were reported to form relatively more compact micelles compared to their linear analogs [14,18,19,25,26,27,28]. Moreover, a higher thermal stability was observed for the micelles of cyclic block copolymer amphiphiles [18,25,26]. Nevertheless, direct structural details on such compact and thermally stable micelles are yet to be given. In fact, a polymer micelle as a platform for application in nanotechnology is highly desirable since it is possible to tailor various physical and chemical properties from the molecular design of the polymer [29,30]. Therefore, the incorporation of cyclic topology in block copolymers for micelle formation began with the goal of achieving an effective polymer micelle system that retains and exhibits the intended properties within the intended size, shape, and structural integrity. 

Such criteria bring light to the great challenge in the development of such a system, which is the difficulty in structural characterization. Dynamic light scattering (DLS), transmission electron microscopy (TEM), and fluorescence/UV-vis spectroscopy are the most popular methods for characterizing the micellar structure but they are limited to providing a few low-resolution structural parameters and rudimentary qualitative insights. Synchrotron X-ray scattering is another option for parameterizing the micellar structure. A quantitative investigation using the technique was reported for the micelle formations of amphiphilic cyclic poly(*n*-butyl acrylate-*b*-ethylene oxide) and its linear triblock analog [31]. The structural analysis presented in the report, however, was limited by an assumption that all micelles were a two-phase sphere in shape; moreover, the report could not provide information on size distributions of the micelles. The utilization of a conventional two-phase sphere modeling approach could not fully realize the micellar structure with high precision. There were more reports utilizing a two-phase sphere modeling approach [32,33,34] but the outcomes of the analyses were faced with a reoccurring lack of precision. Recently, a more quantitative analysis based on a three-phase ellipsoid modeling approach was introduced to examine polymer micelles [35]. Overall, the current progress of the physicochemical investigation on cyclic polymer micelles could be considered to be in the developing stages. Furthermore, the correlation among micellar morphology, polymer chain structure, and physical and chemical properties of the polymer remains to be explored in depth. 

**Figure 1:**
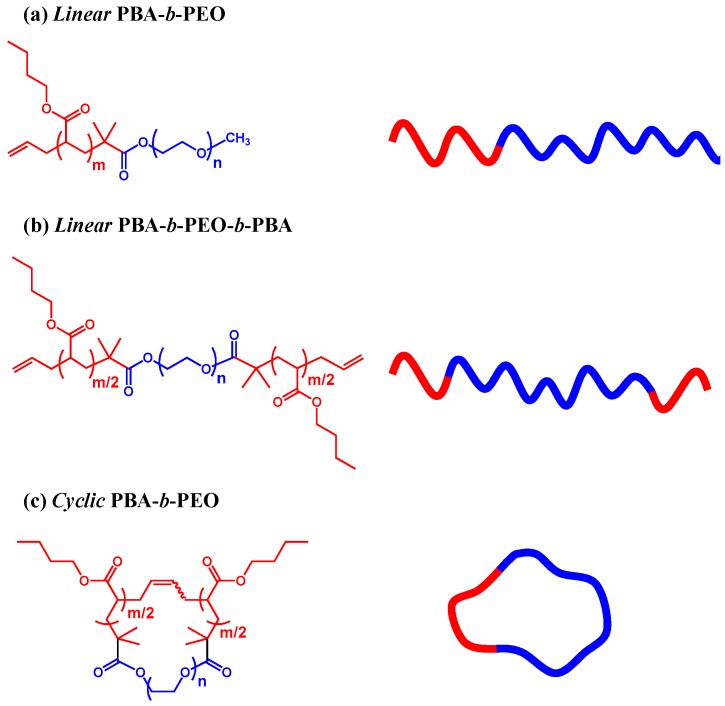
Chemical structures of the amphiphilic block copolymers used in this study.

This paper presents a quantitative analysis on the micellar morphologies of a series of amphiphilic cyclic block copolymers and its linear analogs in a comprehensive manner by using synchrotron X-ray scattering and a combination of data analytical methods: cyclic poly(*n*-butyl acrylate-*b*-ethylene oxide) (*c*-PBA-*b*-PEO) and its linear diblock and triblock analogs (*l*-PBA-*b*-PEO and *l*-PBA-*b*-PEO-*b*-PBA), see Figure 1 and Table 1. The comprehensive scattering data analysis provides new, better insights into the micelles of the topological polymers. Interestingly, in water, the cyclic copolymer and its linear triblock analog form oblate-ellipsoidal micelles and, in contrast, the linear diblock analog creates a prolate-ellipsoidal micelle; essentially, they consist of a core and a solvated corona. For each micellar system, structural parameter details are determined. The parameters provide direct clues for the structural stability of a micelle. High stability of a micelle is achieved by a high core density, a thin and sharp core-corona interface, a low corona density, a thick soft corona shell, a short blob size, a low aggregation number, and a narrow size distribution. The micelle is found to be in the decreasing order of size as *l*-PBA-*b*-PEO >> *l*-PBA-*b*-PEO-*b*-PBA > *c*-PBA-*b*-PEO and in the increasing order of structural stability (i.e., dimensional stability) as *l*-PBA-*b*-PEO << *l*-PBA-*b*-PEO-*b*-PBA < *c*-PBA-*b*-PEO.

## 2. Materials and Methods

### 2.1. Materials

An amphiphilic linear diblock copolymer, poly(*n*-butyl acrylate)-*b*-poly(ethylene oxide) (*l*-PBA-*b*-PEO) was synthesized through the atom transfer radical polymerization (ATRP) of *n*-butyl acrylate (BA) using an *α*-methoxy-*ω*-2-bromoisobutyryl-poly(ethylene oxide) (PEO-1) macroinitiator and subsequent Keck allylation, according to the method reported previously [26,36]. In a similar manner, a linear triblock copolymer analogue, poly(*n*-butyl acrylate)-*b*-poly(ethylene oxide)-*b*-poly(*n*-butyl acrylate) having two allyl end groups (*l*-PBA-*b*-PEO-*b*-PBA) was synthesized by the polymerization of BA with the aid of *α*,*ω*-bis(2-bromoisobutyryl) poly(ethylene oxide) (PEO-2) macroinitiator and subsequent allylation [31]. A cyclic diblock copolymer, poly(*n*-butyl acrylate)-*b*-poly(ethylene oxide) (*c*-PBA-*b*-PEO) was prepared by the cyclization of *l*-PBA-*b*-PEO-*b*-PBA [31]. The synthesis and characterization details are available in the Supplementary Information. The chemical structures and characterization results of the obtained block copolymers are shown in Figure 1 and Table 1.

### 2.2. Micelle Formation

The amphiphilic block copolymer micelles were prepared through a reported method [26,31,36]. Each of the block copolymers was initially dissolved in THF to achieve a polymer solution with a concentration of 10 mg/mL. Then, deionized distilled water was added in a drop-wise manner to the polymer solution under vigorous stirring. Thereafter, the THF was removed from the resulting polymer solution using a rotary evaporator. The resulting micellar solution was filtered using a disposable syringe equipped with a cellulose acetate filter with a pore size of 0.2 μm. For the micellar solutions prepared, the concentration ranged from 0.1 to 0.5 wt %.

### 2.3. Synchrotron X-ray Scattering Measurements

Synchrotron X-ray scattering measurements were conducted at the 4C beamline [37,38] of the Pohang Accelerator Laboratory (PAL). The scattering data of the micellar solution of *l*-PBA-*b*-PEO, *l*-PBA-*b*-PEO-*b*-PBA, and *c*-PBA-*b*-PEO were measured at a sample-to-detector distance (SDD) of 0.5, 1.0, 3.0, and 4.0 m at 25 °C with an X-ray radiation source of *λ* = 0.069 nm or 0.0734 nm (wavelength). All scattering data were collected through a two-dimensional (2D) charge-coupled detector (CCD) (model Rayonix 2D SX 165, Rayonix, Evanston, IL 60201, USA). The scattering angle was calibrated with precalibrated polystyrene-*b*-polyethylene-*b*-polybutadiene-*b*-polystyrene block copolymer and silver behenate powder (Tokyo Chemical Industry (TCI), Tokyo, Japan). The 2D scattering data were circularly averaged with respect to the beam center and normalized to the intensity of the transmitted X-ray beam monitored via a scintillation counter positioned behind the sample. The scattering data were further corrected for the scattering due to the solvent. The representative X-ray scattering intensity *I*(*q*) profiles of the micelles formed by the amphiphilic block copolymers are available in the Supplementary Information (Figure S3).

## 3. Results and Discussion

All measured X-ray scattering data were attempted to be analyzed in a comprehensive, quantitative manner to obtain accurate and precise information regarding micelle shape, stability, size distribution, and critical correlations between polymer topology and micellar structural parameters. With all possible and available analysis schemes considered, spherical core-fuzzy shell (CFS) model analysis [31,39,40,41,42,43], two-phase ellipsoid model analysis [35], three-phase ellipsoid model analysis [35], and Kratky analysis [44] were chosen for this study; in addition, the Guinier method [39,45] and the indirect Fourier transform (IFT) method [39,40,46] were considered. The details of these analysis approaches are available in the Supplementary Information.

**Figure 2:**
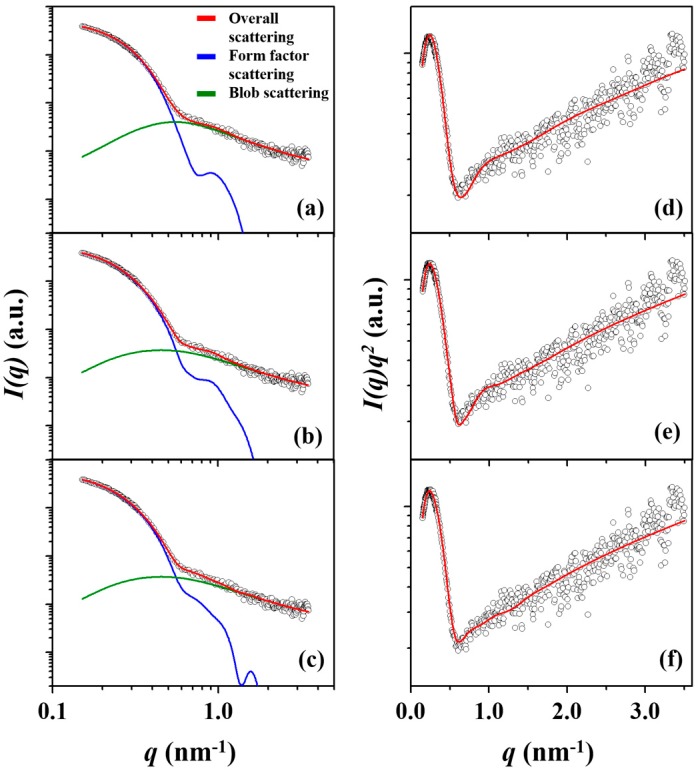
A representative X-ray scattering intensity *I*(*q*) profile and data analysis results of the *l*-PBA-*b*-PEO micelle formed in deionized water with a concentration of 0.5 wt % at 25 °C, which were performed using (**a**) spherical CFS model, (**b**) two-phase ellipsoid model, and (**c**) three-phase ellipsoid model. (**d**–**f**) Kratky representations of the scattering data of the *l*-PBA-*b*-PEO micelle fitted by spherical CFS, two-phase ellipsoid, and three-phase ellipsoid models, respectively. In each figure, the open dot symbols are the measured data and the red solid line represents the sum of the corresponding analysis profile (blue line) and blob contribution (green line). Here, *q* = (4π/*λ*)sin*θ* in which 2*θ* is the scattering angle and *λ* is the wavelength of the X-ray beam used.

In Figure 2a–c, the scattering data of the *l*-PBA-*b*-PEO micelle is fitted by spherical CFS, two-phase ellipsoid, and three-phase ellipsoid models. Each of the three models also accounts for the blob contributions, which are density fluctuations occurring by the dynamic interactions between hydrophilic PEO chain segments and the surrounding solvent molecules of water. Furthermore, the individual scattering profiles reveal a *q*^−2^ dependency in the intermediate-*q* region and a *q*^−5/3^ dependency in the high-*q* region (*q* > 1.8 nm^−1^) regardless of the copolymer topologies (see Kratky plots in Figure 2d–f); *q* = (4π/*λ*)sin*θ* in which 2*θ* is the scattering angle and *λ* is the wavelength of the X-ray beam used. These *q*-dependencies reveal that the hydrophilic PEO blocks in contact with water exhibit a certain level of flexibility and a fuzzy interface with the water medium.

**Figure 3:**
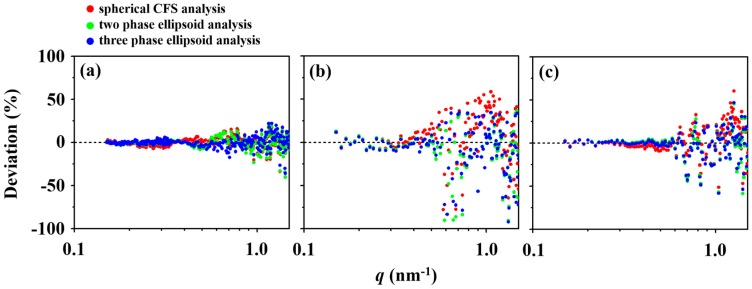
Deviation between the measured scattering data and the data analysis results obtained by various analysis schemes over the *q*-range of 0.15 to 1.5 nm^−1^: (**a**) *l*-PBA-*b*-PEO micelle; (**b**) *l*-PBA-*b*-PEO-*b*- PBA micelle; (**c**) *c*-PBA-*b*-PEO micelle. Each value of deviation was calculated by the following formula [(*I*_obs_ − *I*_calc_)/*I*_obs_] × 100% where *I*_obs_ is the measured scattering intensity and *I*_calc_ is calculated scattering intensity from data analysis. The colored circles represent the deviations from results of spherical CFS (red circles), two-phase ellipsoid (green circles), and three-phase ellipsoid (blue circles) models. The deviations in *q*-range of 1.5 to 3.5 nm^−1^ are not shown due to the insignificantly large deviations arising from the high levels of noise in the raw scattering data in high-*q* regions.

The spherical CFS model analysis presents some deviations from the measured scattering profile over the *q*-range of 0.5 to 1.0 nm^−1^, see Figure 2a,d. The two-phase ellipsoid model analysis presents a similar degree of deviation in the same *q*-range, see Figure 2b,e, in which the deviations are more apparent in the Kratky plot. In contrast, such deviations are no longer observed in the three-phase ellipsoid model analysis, see Figure 2c,f. Figure 3a summarizes the percentage deviation between the measured scattering data and the model analyses, in which the degree of deviation increases in the order from the three-phase ellipsoid, two-phase ellipsoid, to the spherical CFS models. The degree of deviation directly diminishes the precision of the obtained structural parameters and, therefore, it is crucial to achieve model analysis with the lowest possible degree of deviation. All structural parameters obtained from analyzing the measured scattering data of the *l*-PBA-*b*-PEO micelle via spherical CFS, two-phase ellipsoid, and three-phase ellipsoid models are summarized in Table 2.

A substantial deviation from a spherical shape is also detected in the pair distribution function (*p*(*r*)) profile of IFT analysis, a model independent method. Theoretically, the *p*(*r*) function of an ideal sphere should take on a symmetric bell shape but the *p*(*r*) function of *l*-PBA-*b*-PEO micelle reveals a significantly right-skewed shape, see Supplementary Information, Figure S1a. The IFT analysis provides other structural parameters, see Supplementary Information, Table S1: *R*_g,IFT_ (radius of gyration), *D*_max_ (maximum dimension or maximum diameter), *R*_max_ (radius of micelle at the peak maximum), and *R*_max_/*R*_g,IFT_ ratio. An ideal sphere should reveal *D*_max_/*R*_max_ = 2, in which its radius is exactly half of its diameter, and *R*_max_/*R*_g,IFT_ = 1.36; however, *D*_max_/*R*_max_ = 3.45 and *R*_max_/*R*_g,IFT_ = 1.09 for the *l*-PBA-*b*-PEO micelle. The *R*_g,IFT_ (7.63 nm) is slightly different from that (7.58 nm *R*_g,G_) obtained by the Guinier analysis assuming an ideal sphere, see Supplementary Information, Table S1 and Figure S2a. Therefore, the spherical CFS model is inadequate for describing a non-spherical micelle and the resulting structural parameters contain low accuracy and significant levels of error. The relatively high degree of deviation, shown in Figure 3a, should also indicate low precision in the structural parameters. Specifically, the radius *R*_e,micelle_, corona thickness *t*_corona_, and radius of gyration *R*_g_ of micelle are underestimated while the core radius *r*_core_, fuzzy corona thickness *t*_f,corona_, blob radius *ξ*, density of corona *ρ*_corona_, and aggregation number of block copolymer chains *N*_agg_ of micelle are overestimated. The two-phase ellipsoid model, on the contrary, is capable of describing a non-spherical micelle and, therefore, improves the accuracy of the structural parameters. Figure 3a shows, however, that the degree of deviation for a two-phase ellipsoid model is only slightly less than the spherical CFS model for a *q*-range above 0.5 nm^−1^, which indicates that the precision of parameters is still lacking. This is reflected on the noticeably low density of corona *ρ*_corona_ and low value of *R*_g_. Conceptually, it is rather difficult to describe the corona region of a micelle with a single phase due to the rapid decay in electron density, see Supplementary Information, Figure S1b. With the addition of another phase, however, the three-phase ellipsoid model could overcome this issue. It is shown that the three-phase ellipsoid model analysis provides the best fit of the measured scattering data with the lowest degree of deviation. The resulting set of structural parameters, see Table 2, is the most realistic, accurate, and precise compared to the other sets provided by spherical CFS and two-phase ellipsoid models. Thus, *l*-PBA-*b*-PEO is confirmed to form micelles with large prolate ellipsoid geometry rather than a spherical shape from this series of quantitative scattering data analyses.

**Figure 4:**
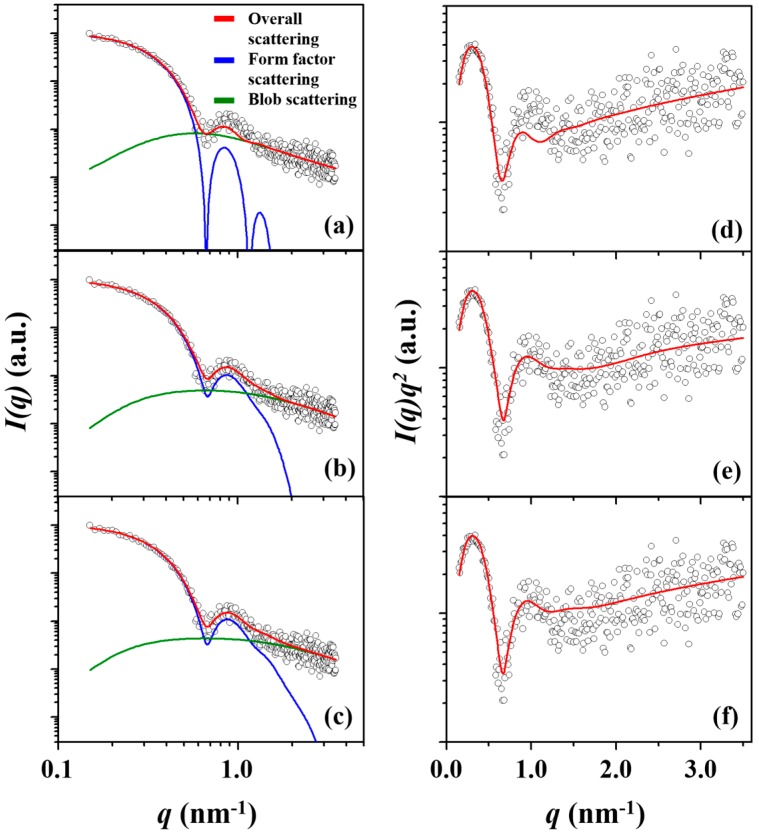
A representative X-ray scattering intensity *I*(*q*) profile and data analysis results of the *l*-PBA-*b*-PEO-*b*-PBA micelle formed in deionized water with a concentration of 0.5 wt % at 25 °C, which were performed using (**a**) a spherical CFS model, (**b**) a two-phase ellipsoid model, and (**c**) a three-phase ellipsoid model. (**d**–**f**) Kratky representations of the scattering data of the *l*-PBA-*b*-PEO micelle fitted by spherical CFS, two-phase ellipsoid, and three-phase ellipsoid models, respectively. In each figure, the open dot symbols are the measured data and the red solid line represents the sum of the corresponding analysis profile (blue line) and blob contribution (green line). *q* = (4π/*λ*)sin*θ* in which 2*θ* is the scattering angle and *λ* is the wavelength of the X-ray beam used.

The same set of the three different model analyses were applied to the measured scattering data of the *l*-PBA-*b*-PEO-*b*-PBA micelle. Figure 4a–c shows the data fitting results of the individual models with blob contributions, and the conversion into Kratky plot displays *q*^−2^ and *q*^−5/3^ dependencies in the intermediate and high-*q* regions, respectively, see Figure 4d–f. Such characteristics indicate density fluctuations originating from the flexible hydrophilic blocks in the micelle corona and the presence of a fuzzy interface between the corona and the solvent.

The result of spherical CFS analysis, see Figure 4a,d, shows deviations from the measured scattering data over the *q*-range 0.7–1.5 nm^−1^. In Figure 3, it is indicated that the degree of deviation for the analysis of the *l*-PBA-*b*-PEO micelle is lesser than *l*-PBA-*b*-PEO-*b*-PBA micelle. Although the level of noise in the scattering data of the *l*-PBA-*b*-PEO-*b*-PBA micelle is higher than that of the *l*-PBA-*b*-PEO micelle, the presence of an evident mismatch between the calculated scattering curve and the measured data denies the adequacy of a spherical CFS model for reliable analysis. The origin of a spherical CFS model’s limitation, once again, stems from the non-spherical shape of the *l*-PBA-*b*-PEO-*b*-PBA micelle that was identified in the IFT analysis, see Supplementary information, Figure S1a and Table S1. Nevertheless, the previous study analyzed the scattering data by adopting three different analysis schemes (Guinier, spherical CFS, and spherical copolymer micelle model approaches) that all assume the micelle to be spherical [31]. Figure 4b,c and Figure 4e,f, however, show both two-phase and three-phase ellipsoid models are able to fit the measured data more effectively, with the three-phase ellipsoid model analysis having the least deviation and best fit overall, see Figure 3b. All sets of obtained structural parameters are summarized in Table 3. The spherical CFS analysis overestimates all structural parameters, compared to those determined by the three-phase ellipsoid analysis. The two-phase ellipsoid analysis also overestimates *R*_e,micelle_, *r*_core_, *ξ*, and *N*_agg_; however, *t*_corona_, *ε* (=polar radius/equatorial radius, ellipsoidicity ratio), *R*_g_, and *ρ*_corona_ are underestimated. Overall, detailed analyses found that the *l*-PBA-*b*-PEO-*b*-PBA micelle has an oblate ellipsoid shape rather than spherical shape and prolate ellipsoid geometry.

**Figure 5:**
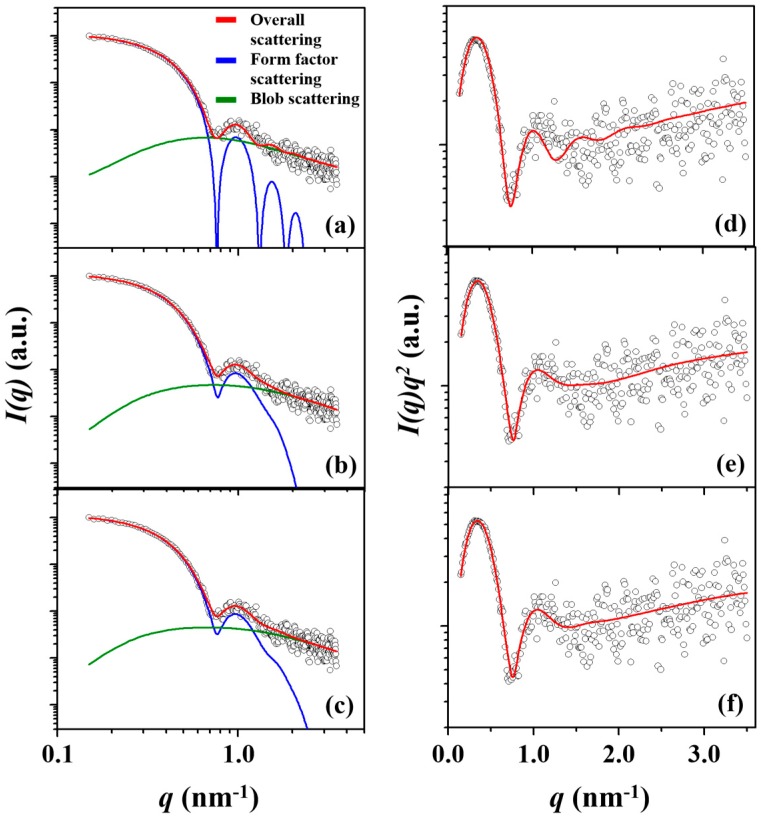
A representative X-ray scattering intensity *I*(*q*) profile and data analysis results of the *c*-PBA-*b*-PEO micelle formed in deionized water with a concentration of 0.5 wt % at 25 °C, which were performed using (**a**) spherical CFS model, (**b**) two phase ellipsoid model, and (**c**) three phase ellipsoid model. (**d**–**f**) Kratky representations of the scattering data of the *l*-PBA-*b*-PEO micelle fitted by spherical CFS, two phase ellipsoid, and three phase ellipsoid models, respectively. In each figure, the open dot symbols are the measured data and the red solid line represents the sum of the corresponding analysis profile (blue line) and blob contribution (green line). *q* = (4π/*λ*)sin*θ* in which 2*θ* is the scattering angle and *λ* is the wavelength of the X-ray beam used.

The scattering data of *c*-PBA-*b*-PEO micelle were analyzed in the same manner as other copolymer micelles. The analyses found that the three phase ellipsoid model most successfully fits the scattering data, compared to the other two methods (Figure 5a–c; Figure 3c). The *q* dependency characteristic is similar to those of other copolymer micelles where blob contributions in the measured scattering data occur due to the flexible nature of hydrophilic PEO blocks within the micelle corona, and the presence of fuzzy interface between the micelle corona and the solvent medium. The structural parameters from three phase ellipsoid model analysis (summarized in Table 4) inform that *c*-PBA-*b*-PEO micelle also takes the shape of an oblate ellipsoid similar to *l*-PBA-*b*-PEO-*b*-PBA micelle. The spherical CFS model analysis, similar to aforementioned results, provides erroneous parameters by underestimating *R*_e,micelle_ and *t*_corona_ and overestimating *r*_core_, *ξ*, *R*_g_, *ρ*_corona_, and *N*_agg_. The two phase ellipsoid model analysis also underestimates *R*_e,micelle_, *t*_corona_, *ε*, *R*_g_, and *ρ*_corona_; but *r*_core_, *ξ*, and *N*_agg_ are overestimated.

For the micelles of the cyclic block copolymer and its linear analogs, the results of the most appropriate three-phase ellipsoid model analysis are summarized in Table 5. These structural parameters are examined and compared in detail to comprehend the correlations between the various aspects of the molecular topology and micellar morphology.

Firstly, all block copolymers form micelles based on the two essential components: a core and a corona. The formation of a core-corona micelle is driven by the solvent-affinity difference between the PBA and PEO blocks where PEO exhibits a higher affinity to water than PBA. Therefore, the micelle core is always formed by the PBA block while the micelle corona consists of the PEO block, regardless of the molecular topologies.

Secondly, both *l*-PBA-*b*-PEO-*b*-PBA and *c*-PBA-*b*-PEO form micelles with an oblate ellipsoid geometry while the *l*-PBA-*b*-PEO micelle forms a prolate ellipsoid shape. Interestingly, both *l*-PBA-*b*-PEO-*b*-PBA and *l*-PBA-*b*-PEO possess a linear topology but the micellar shape is drastically different. Due to the geometrical confinement associated with the block connectivities in a triblock copolymer configuration, the hydrophilic PEO block could be accommodated into the corona formation by having a loop shape. As a result, both the *l*-PBA-*b*-PEO-*b*-PBA micelle and the *c*-PBA-*b*-PEO micelle have corona components that do not possess molecular chain ends. This is a major difference between the *l*-PBA-*b*-PEO micelle versus the *l*-PBA-*b*-PEO-*b*-PBA micelle and *c*-PBA-*b*-PEO micelle, in which the loop formation of the hydrophilic PEO blocks residing in the corona becomes the critical factor determining the shape of the micelle. In addition, it is noted that the *c*-PBA-*b*-PEO micelle exhibits a slightly higher degree of oblate character (i.e., slightly lower *ε* value) than the *l*-PBA-*b*-PEO-*b*-PBA micelle. This might be attributed to the slightly larger thickness of its solvated corona (*t*_s.corona_) despite the smaller core radius (*r*_core_) and lower aggregation number (*N*_agg_). 

Thirdly, the *c*-PBA-*b*-PEO micelle has unique features that distinguish itself from the *l*-PBA-*b*-PEO-*b*-PBA micelle and *l*-PBA-*b*-PEO micelle; higher core density *ρ*_core_ and sharper core-corona interface *t*_f__,core_ than those of linear analog micelles. Considering the cyclic topology of *c*-PBA-*b*-PEO, the hydrophobic PBA would participate in the micelle core formation as a folded chain formation. This unique chain conformation is free from the entropic disturbance that may arise from a chain end and, therefore, is able to exhibit an aggregation behavior with relatively higher density than that of a simple linear chain conformation. Furthermore, a denser micelle core should naturally have a sharper core-corona interface since the increased core density would establish a more defined boundary where the solvent molecules are unable to penetrate into. Considering this fact, the structural parameters regarding the micelle core inform that the participation of the PBA block with a folded chain conformation is correlated to the formation of a denser and more stable micelle core. In general, the polymer chains constituting the micelle corona are relatively flexible and mobile due to the dynamic interactions with solvent molecules; thus, the overall structural stability of the micelle may be governed primarily by the core stability. Considering this point, the participation of the PBA block with a folded chain conformation could greatly contribute to the overall stability of the micelle in addition to the core stability. Namely, the cyclic topology brings a positive impact to the overall structural stability of polymer micelle.

Fourthly, the core-corona interface of the *l*-PBA-*b*-PEO-*b*-PBA micelle is less sharp than that of the cyclic analog micelle but sharper than that of the linear diblock analog micelle. For both linear analogs, the PBA blocks participate in the core formation with linear chain conformations rather than a folded conformation. Considering these facts, the stability of the core in the *l*-PBA-*b*-PEO-*b*-PBA micelle could be contributed by the loop chain formation of the PEO block in the micelle corona. As a result, the overall structural stability of the *l*-PBA-*b*-PEO-*b*-PBA micelle is relatively higher than that of the *l*-PBA-*b*-PEO micelle.

Fifthly, *c*-PBA-*b*-PEO requires the relatively lowest aggregation number *N*_agg_ to form a micelle, whereas its linear diblock analog requires the highest *N*_agg_. The *N*_agg_ is in the increasing order of: *c*-PBA-*b*-PEO micelle < *l*-PBA-*b*-PEO-*b*-PBA micelle << *l*-PBA-*b*-PEO micelle. The lowest *N*_agg_ of *c*-PBA-*b*-PEO is another indication of high structural stability (i.e., dimensional stability). As a consequence, the highest *N*_agg_ of *l*-PBA-*b*-PEO indicates the lowest structural stability. This rationale is based on the fact that micelle formation is driven by the solvent-affinity difference between the PBA and PEO blocks. In other words, hydrophobic PBA blocks are highly unstable when surrounded by water molecules, and an aggregation amongst PBA chains from nearby copolymers is triggered to form a micelle core. The cohesive energy (i.e., enthalpic gain) from the PBA chain aggregation inside the core thereby stabilizes the core itself from the previously energetically unfavorable state. However, it is noteworthy that an increased number of PBA chains involved in the micelle core aggregation behavior would also increase the entropy within the core. Hence, the notion of lower *N*_agg_ leading to a more stable micelle core is established. For the *l*-PBA-*b*-PEO micelle, the high *N*_agg_ may be a consequence of the relatively longer length of the PEO block in comparison to those in the cyclic and triblock analogs because the larger amount of dynamic interactions with water molecules may feedback to lower the core’s stability.

Sixthly, all micelles of this study consist of basically two main parts, namely the core and the corona. Therefore, an appropriate balance in length scales of core and corona parts could directly correlate to the micelle stability. Compared to the core radii, the corona thickness is 3.47 times larger for the *l*-PBA-*b*-PEO micelle, 2.62 times larger for the *l*-PBA-*b*-PEO-*b*-PBA micelle, and 2.67 times larger for the *c*-PBA-*b*-PEO micelle. The thicker corona of *l*-PBA-*b*-PEO micelle indicates a larger amount of water molecules actively interacting with the PEO blocks than the *l*-PBA-*b*-PEO-*b*-PBA micelle and *c*-PBA-*b*-PEO micelle. Then, it is logical to assume that the aforementioned structural stabilization involved in the micelle core formation would additionally have to account for the dynamic interactions from the corona that negatively feedbacks to the overall structural stability. As a result, a larger imbalance of corona thickness to core radius might destabilize the micellar structure.

Seventhly, the corona of *c*-PBA-*b*-PEO micelle reveals *ρ*_corona_ = 0.058 g/cm^3^ (density of corona). This *ρ*_corona_ value is nearly identical to that (0.059 g/cm^3^) of the *l*-PBA-*b*-PEO micelle. However, the corona of *c*-PBA-*b*-PEO micelle has quite different structural characteristics in comparison to that of the *l*-PBA-*b*-PEO micelle. The thickness of the corona is 4.40 nm, which is 33.3% thinner than that (6.60 nm) of the *l*-PBA-*b*-PEO micelle. Moreover, such a thin corona shell consists of a soft sublayer (i.e., less dense and more flexible) sublayer as a major component (86.4%) and a dense sublayer as a minor component (13.6%). Due to these structural characteristics, the micelle corona exhibits a very small blob radius (*ξ* = 0.60 nm, average correlation length of density fluctuation). In contrast, the corona shell of the *l*-PBA-*b*-PEO micelle is composed of a thick dense layer (72.7% of the corona) and a thin soft layer (27.3%); as a result, *ξ* = 1.70 nm, which is 2.83 times larger than that of the *c*-PBA-*b*-PEO micelle. These results collectively indicate that, in the *c*-PBA-*b*-PEO micelle, the degree of solvation of hydrophilic PEO blocks is higher than the *l*-PBA-*b*-PEO micelle. Higher solvation would then indicate less densely packed PEO chains that are more flexible despite the looped chain conformation arising from the cyclic topology. Overall, it is very interesting that the PEO block of *c*-PBA-*b*-PEO behaves with a larger hydrodynamic volume to override any penalties potentially originating from the looped chain conformation. Due to this large hydrodynamic volume, the PEO blocks can effectively fill the volume taken by the micelle corona, protecting the micelle from any undesired secondary aggregations with nearby micelles. Consequently, the topology-induced large hydrodynamic volume character of the PEO block makes a large contribution towards raising the stability of *c*-PBA-*b*-PEO micelle. Similar features are observed on the PEO block in the *l*-PBA-*b*-PEO-*b*-PBA micelle, see Table 5. Surprisingly, the PEO block of *l*-PBA-*b*-PEO can be interpreted to be less solvated, despite having a linear chain conformation and the chain end. The notion of less solvated linear PEO blocks is counterintuitive but the high *N*_agg_ value indicates a high number of PEO blocks constituting the micelle corona. Then, the involvement of more PEO blocks would lead to a lower level of solvation in comparison to the *c*-PBA-*b*-PEO micelle. Namely, the lower solvation and increased chain rigidity characteristics of the PEO block could cause a negative contribution to the corona stability of the *l*-PBA-*b*-PEO micelle.

**Figure 6:**
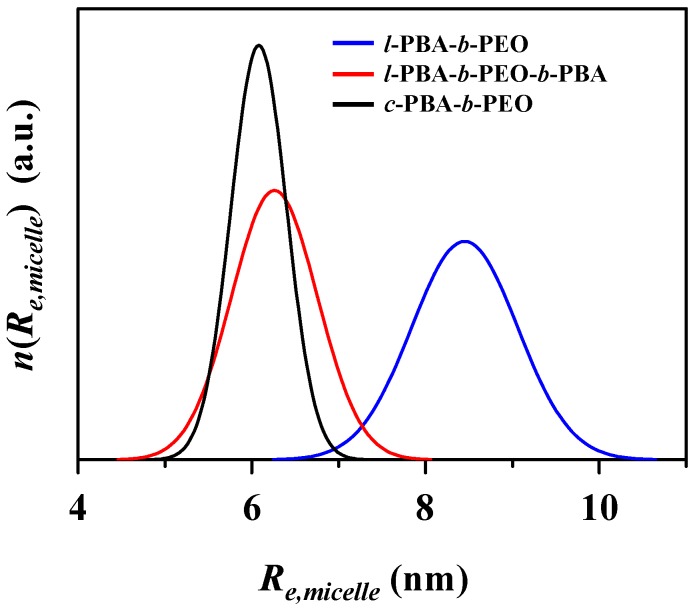
Size distribution curves of the micelles of topological block copolymers which are expressed based on the equatorial radius *R*_e,micelle_ of micelles extracted by the scattering data analyses using a three-phase ellipsoid scheme.

Eighthly, the three-phase ellipsoid analysis provides the micelle size distribution. As shown in Figure 6, the size (i.e., radius) distribution is in the increasing order, *c*-PBA-*b*-PEO micelle < *l*-PBA-*b*-PEO-*b*-PBA micelle << *l*-PBA-*b*-PEO micelle. These size distributions are directly correlated to the overall structural stabilities of the individual micelle systems in which higher structural stability reveals narrower size distribution. The results support that the cyclic topology is indeed beneficial in establishing block copolymer micelles with good structural stability.

**Figure 7:**
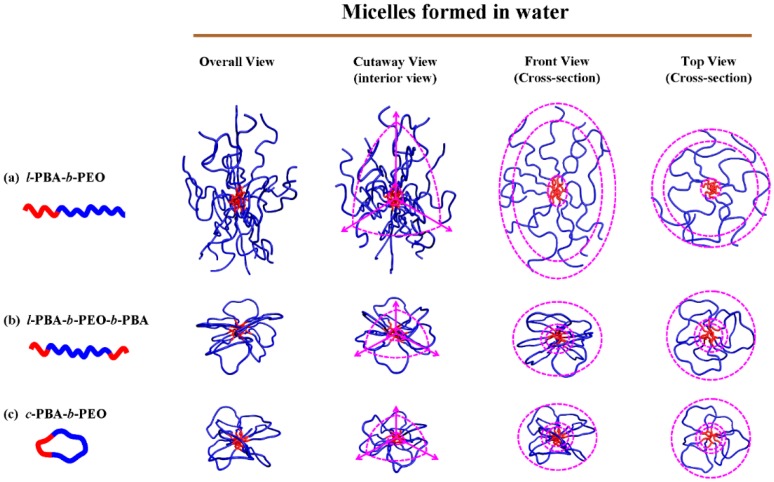
Schematic representations of the micelle structures of amphiphilic topological block copolymers based on the structural parameters obtained from the quantitative X-ray scattering data analyses using a three-phase ellipsoid scheme.

Lastly, the micellar structures of the amphiphilic cyclic diblock copolymer and its linear diblock and triblock analogs have been constructed schematically based on the structural parameters in Table 5. The constructed micellar structures are compared in Figure 7.

As discussed above, the micelle formation, structural stability, and all the structural parameters of an amphiphilic block copolymer are governed by the molecular topology with significance. Overall, a cyclic block copolymer is quite beneficial to make the most compact, highly stable micelle that attains a narrow size distribution.

## 4. Conclusions

In this study, the micelles of a cyclic diblock copolymer amphiphile and its linear diblock and triblock analogs in water have been investigated in detail using synchrotron X-ray scattering and data analysis in a comprehensive manner; *c*-PBA-*b*-PEO micelle, *l*-PBA-*b*-PEO-*b*-PBA micelle, and *l*-PBA-*b*-PEO micelle. From this comprehensive scattering analysis, we have gained new insights into the micelles made of the amphiphiles in three different topologies as follows.

All block copolymers form stable micelles consisting of a PBA-based core and a PEO-based corona, and their shapes are ellipsoidal rather than spherical. However, the cyclic polymer and its triblock analog micelles reveal oblate ellipsoid shapes with small ellipsoidicity ratios, whereas the linear diblock analog micelle shows prolate ellipsoid shape with a large ellipsoidicity ratio.

The stability and structural characteristics of the micelles are significantly dependent upon the topologies of amphiphiles. The micellar structural stability is in the increasing order *l*-PBA-*b*-PEO << *l*-PBA-*b*-PEO-*b*-PBA < *c*-PBA-*b*-PEO.

Factors that lead to highly stable micelles could be obtained directly from its structural parameters: (i) core density (*ρ*_core_), (ii) core-corona interface thickness (i.e., *t*_f,core_, interfacial thickness of core and dense corona), (iii) corona density (*ρ*_corona_), (iv) ratio of dense and soft corona thicknesses (*t*_d,corona_/*t*_f,s,corona_), (v) correlation length of density fluctuation (i.e., blob radius) in the entire corona region (*ξ*), (vi) polymer chain aggregation number (*N*_agg_), and (vii) size distribution. Higher stability of the micelle is architecturally supported by a larger *ρ*_core_, thinner *t*_f,core_, lower *ρ*_corona_, smaller *t*_d,corona_/*t*_f,s,corona_ ratio, shorter *ξ*, lower *N*_agg_, and narrower size distribution.

In summary, this study has provided insights that suggest that the overall structural stability of micelle could be primarily governed by the formation of a highly dense core with high enthalpy gain (i.e., high cohesive energy) and secondarily supported by the formation of the corona assembled with chains exhibiting a large hydrodynamic volume with high enthalpy and entropy gains (i.e., a high solvent interaction and high chain mobility). Owing to the large hydrodynamic volume, the hydrophilic blocks associated in the micelle corona can easily fill the corona’s volume, effectively preventing any unfavorable secondary aggregations with other micelles. Such architecturally well-defined and highly stable micelles could be demonstrated by the cyclic diblock amphiphile of this study, in which the hydrophobic PBA block can pack densely with a folded chain conformation to form a highly dense core, and the hydrophilic PEO block can be properly solvated with a loop chain conformation with large hydrodynamic volume. A moderate level of stability of the linear triblock analog micelle could be attributed mainly to the hydrophilic PEO block behaving to take the loop chain conformation and a large hydrodynamic volume.

## Figures and Tables

**Table 1:** Molecular characteristics, degree of polymerization, and volume fraction components of the block copolymers used in this study.

Amphiphilic Block Copolymer	${\bar{M}}_{n,\mathrm{NMR}}{}^{a}\;(g/\mathrm{mol})$	*PDI* ^b^	PBA Block		PEO Block	
			${\bar{\mathrm{DP}}}_{\mathrm{PBA}}{}^{c}$	*φ*_PBA_^d^	${\bar{\mathrm{DP}}}_{\mathrm{PEO}}$ ^e^	*φ*_PEO_^f^
*l*-PBA-*b*-PEO	6830	1.07	9	0.177	126	0.823
*l*-PBA-*b*-PEO-*b*-PBA	5850	1.11	10	0.331	69	0.669
*c*-PBA-*b*-PEO	4700	1.18	10	0.334	68	0.666

^a^ Number-average molecular weight of polymer determined by ^1^H NMR spectroscopic analysis. ^b^ Polydispersity index of polymer determined by GPC analysis in THF. ^c^ Number-average degree of polymerization of PBA block determined by ^1^H NMR spectroscopy. ^d^ Volume fraction of the PBA block estimated from the values of ${\bar{M}}_{n,\mathrm{NMR}}$ and mass density *ρ*_m_. ^e^ Number-average degree of polymerization of the PEO block determined by ^1^H NMR spectroscopy. ^f^ Volume fraction of the PEO block estimated from the values of ${\bar{M}}_{n,\mathrm{NMR}}$ and mass density *ρ*_m_. The *ρ*_m_ value was 1.08 g/cm^3^ for linear PBA homopolymer and 1.12 g/cm^3^ for linear PEO homopolymer [31].

**Table 2:** Structural parameters of the *l*-PBA-*b*-PEO micelle obtained from the quantitative analysis of scattering data with various model approaches.

Structural Parameter				*l*-PBA-*b*-PEO Micelle		
				Spherical CFS	Two-Phase Ellipsoid	Three-Phase Ellipsoid
*R*_e,micelle_^a^ (nm)				7.10	7.20	8.50
	*r*_core_^b^ (nm)			2.80	2.10	1.90
		*t*_f__,core_^c^ (nm)			1.00	0.50
		*t*_corona_^d^ (nm)		4.30	5.10	6.60
		*t*_f.corona_^e^ (nm)		3.66	2.55	
			*t*_d,corona_^f^ (nm)			4.80
			*t*_f__,d.corona_^g^ (nm)			0.72
			*t*_s.corona_^h^ (nm)			1.80
			*t*_f__,s.corona_^i^ (nm)			0.90
*ε* ^j^				1.0	1.5	1.5
*ξ*^k^ (nm)				3.00	1.70	1.70
*R*_g_^l^ (nm)				5.50	6.64	7.84
*ρ*_core_^m^ (g/cm^3^)				1.08	1.08	1.08
*ρ*_corona_^n^ (g/cm^3^)				0.348	0.013	0.059
*N*_agg_^o^				51.9	32.9	24.3

^a^ Micelle radius in the equatorial direction. ^b^ Radius of the core. ^c^ Thickness of the fuzzy part (interfaced with the dense corona) of the core. ^d^ Radius of the corona. ^e^ Thickness of the fuzzy part (interfaced with solvent) of the corona. ^f^ Thickness of dense corona. ^g^ Thickness of the fuzzy part (interfaced with the soft corona) of the dense corona. ^h^ Thickness of the solvated corona. ^i^ Thickness of the fuzzy part (interfaced with solvent) of the solvated corona. ^j^ Ellipsoidicity ratio (*ε* = polar radius/equatorial radius). ^k^ Average correlation length of density fluctuation (i.e., blob radius) in the entire corona region. ^l^ Radius of gyration of micelle. ^m^ The density of micelle core is assumed to be the density of linear PBA homopolymer in films. ^n^ Density of corona estimated from the aggregation number of copolymer chains *N*_agg_, the number-average molecular weight of the PEO block, and the total volume of the corona. ^o^ Aggregation number of copolymer chains in a single micelle, which was estimated from the core volume and the assumption that the density of the core composed of PBA block is same with that of the corresponding *ρ*_core_ values.

**Table 3:** Structural parameters of the *l*-PBA-*b*-PEO-*b*-PBA micelle obtained from the quantitative analysis of scattering data with various model approaches.

Structural Parameter				*l*-PBA-*b*-PEO-*b*-PBA Micelle		
				Spherical CFS	Two-Phase Ellipsoid	Three-Phase Ellipsoid
*R*_e,micelle_^a^ (nm)				6.50	6.30	6.26
	*r*_core_^b^ (nm)			1.85	2.30	1.73
		*t*_f__,core_^c^ (nm)			0.60	0.40
		*t*_corona_^d^ (nm)		4.65	4.00	4.45
		*t*_f.corona_^e^ (nm)		0.89	1.95	
			*t*_d,corona_^f^ (nm)			0.80
			*t*_f__,d.corona_^g^ (nm)			0.31
			*t*_s.corona_^h^ (nm)			3.65
			*t*_f__,s.corona_^i^ (nm)			1.74
*ε* ^j^				1.0	0.79	0.83
*ξ*^k^ (nm)				1.71	0.70	0.55
*R*_g_^l^ (nm)				5.03	4.56	4.59
*ρ*_core_^m^ (g/cm^3^)				1.08	1.08	1.08
*ρ*_corona_^n^ (g/cm^3^)				0.060	0.013	0.054
*N*_agg_^o^				13.5	20.5	9.1

^a^ Micelle radius in the equatorial direction. ^b^ Radius of the core. ^c^ Thickness of the fuzzy part (interfaced with the dense corona) of the core. ^d^ Radius of the corona. ^e^ Thickness of the fuzzy part (interfaced with solvent) of the corona. ^f^ Thickness of dense corona. ^g^ Thickness of the fuzzy part (interfaced with the soft corona) of the dense corona. ^h^ Thickness of the solvated corona. ^i^ Thickness of the fuzzy part (interfaced with solvent) of the solvated corona. ^j^ Ellipsoidicity ratio (*ε* = polar radius/equatorial radius). ^k^ Average correlation length of density fluctuation (i.e., blob radius) in the entire corona region. ^l^ Radius of gyration of micelle. ^m^ The density of micelle core is assumed to be the density of linear PBA homopolymer in films. ^n^ Density of corona estimated from the aggregation number of copolymer chains *N*_agg_, the number-average molecular weight of the PEO block, and the total volume of the corona. ^o^ Aggregation number of copolymer chains in a single micelle, which was estimated from the core volume and the assumption that the density of the core composed of PBA block is same with that of the corresponding *ρ*_core_ values.

**Table 4:** Structural parameters of the *c*-PBA-*b*-PEO micelle obtained from the quantitative analysis of scattering data with various model approaches.

Structural Parameter				*c*-PBA-*b*-PEO Micelle		
				Spherical CFS	Two-Phase Ellipsoid	Three-Phase Ellipsoid
*R*_e,micelle_^a^ (nm)				5.96	5.75	6.05
	*r*_core_^b^ (nm)			1.69	2.20	1.65
		*t*_f__,core_^c^ (nm)			0.50	0.30
		*t*_corona_^d^ (nm)		4.27	3.55	4.40
		*t*_f.corona_^e^ (nm)		0.64	1.73	
			*t*_d,corona_^f^ (nm)			0.60
			*t*_f__,d.corona_^g^ (nm)			0.12
			*t*_s.corona_^h^ (nm)			3.80
			*t*_f__,s.corona_^i^ (nm)			1.44
*ε* ^j^				1.0	0.78	0.82
*ξ*^k^ (nm)				1.23	0.70	0.60
*R*_g_^l^ (nm)				4.62	4.15	4.42
*ρ*_core_^m^ (g/cm^3^)				1.23	1.23	1.23
*ρ*_corona_^n^ (g/cm^3^)				0.067	0.016	0.058
*N*_agg_^o^				11.7	20.1	8.9

^a^ Micelle radius in equatorial direction. ^b^ Radius of the core. ^c^ Thickness of the fuzzy part (interfaced with the dense corona) of the core. ^d^ Radius of the corona. ^e^ Thickness of the fuzzy part (interfaced with solvent) of the corona. ^f^ Thickness of dense corona. ^g^ Thickness of the fuzzy part (interfaced with the soft corona) of the dense corona. ^h^ Thickness of solvated corona. ^i^ Thickness of the fuzzy part (interfaced with solvent) of the solvated corona. ^j^ Ellipsoidicity ratio (*ε* = polar radius/equatorial radius). ^k^ Average correlation length of density fluctuation (i.e., blob radius) in the entire corona region. ^l^ Radius of gyration of micelle. ^m^ Density value of micelle core consisting of cyclic PBA-*b*-PEO [31]. ^n^ Density of corona estimated from the aggregation number of copolymer chains *N*_agg_, the number-average molecular weight of the PEO block, and the total volume of the corona. ^o^ Aggregation number of copolymer chains in a single micelle, which was estimated from the core volume and the assumption that the density of the core composed of PBA block is same with that of the corresponding *ρ*_core_ values.

**Table 5:** Structural parameters of micelles of the amphiphilic block copolymers obtained from the quantitative analysis of scattering data with three phase ellipsoid model.

Structural Parameter			Amphiphilic Block Copolymer Micelle		
			*l*-PBA-*b*-PEO	*l*-PBA-*b*-PEO-*b*-PBA	*c*-PBA-*b*-PEO
*R*_e,micelle_^a^ (nm)			8.50 (0.61) ^b^	6.26 (0.49)	6.05 (0.32)
	*r*_core_^b^ (nm)		1.90 (0.35)	1.73 (0.25)	1.65 (0.15)
		*t*_f__,core_^d^ (nm)	1.90 (0.35)	1.73 (0.25)	1.65 (0.15)
		*t*_d,corona_^e^ (nm)	0.50	0.40	0.30
		*t*_f__,d.corona_^f^ (nm)	4.80 (0.40)	0.80 (0.15)	0.60 (0.05)
		*t*_s.corona_^g^ (nm)	0.72	0.31	0.12
		*t*_f__,s.corona_^h^ (nm)	1.80 (0.30)	3.65 (0.40)	3.80 (0.10)
*ε* ^i^			1.50	0.83	0.82
*ξ*^j^ (nm)			1.70	0.55	0.60
*R*_g_^k^ (nm)			7.84	4.59	4.42
*ρ*_core_^l^ (g/cm^3^)			1.08	1.08	1.23 ^m^
*ρ*_corona_^n^ (g/cm^3^)			0.059	0.054	0.058
*N*_agg_^o^			24.3	9.1	8.9

^a^ Micelle radius in equatorial direction. ^b^ Standard deviation. ^c^ Radius of the core. ^d^ Thickness of the fuzzy part (interfaced with the dense corona) of the core. ^e^ Thickness of the dense corona. ^f^ Thickness of the fuzzy part (interfaced with the soft corona) of the dense corona. ^g^ Thickness of the solvated corona. ^h^ Thickness of the fuzzy part (interfaced with solvent) of the solvated corona. ^i^ Ellipsoidicity ratio (*ε* = polar radius/equatorial radius). ^j^ Average correlation length of density fluctuation (i.e., blob radius) in the entire corona region. ^k^ Radius of gyration of micelle. ^l^ Density of micelle core is assumed to be the density of linear PBA homopolymer. ^m^ Density value of micelle core consisting of cyclic PBA-*b*-PEO [31]. ^n^ Density of corona estimated from the aggregation number of copolymer chains *N*_agg_, the number-average molecular weight of the PEO block, and the total volume of the corona. ^o^ Aggregation number of copolymer chains in a single micelle, which was estimated from the core volume and the assumption that the density of the core composed of PBA block is same with that of the corresponding *ρ*_core_ values.

## Supplementary Materials

The following are available online at http://www.mdpi.com/2073-4360/11/1/163/s1, Figure S1: (a) Pair distance distribution functions *p*(*r*) and (b) normalized radial density distribution functions *ρ*(*r*) obtained by IFT analysis of the X-ray scattering data, Figure S2: Guinier plots of the X-ray scattering data of micelles: (a) *l*-PBA-*b*-PEO; (b) *l*-PBA-*b*-PEO-*b*-PBA; (c) *c*-PBA-*b*-PEO. The white dot symbols are the measured data and the red solid lines represent the profiles obtained by fitting the data using the Guinier law, Figure S3: Representatives of the X-ray scattering intensity *I*(*q*) profiles measured for the micelles of amphiphilic block copolymers formed in deionized water with a polymer concentration of 0.5 wt % at 25 °C: (a) *l*-PBA-*b*-PEO; (b) *l*-PBA-*b*-PEO-*b*-PBA; (c) *c*-PBA-*b*-PEO. *q* = (4π/*λ*)sin*θ* in which 2*θ* is the scattering angle and λ is the wavelength of the X-ray beam used. Table S1: Structural parameters of micelles of the amphiphilic block copolymers obtained from Guinier and indirect Fourier transform (IFT) analyses.
