# Supplementary material for: Micelle Structure Details and Stabilities of Cyclic Block Copolymer Amphiphile and Its Linear Analogues
Source: Polymers (Basel). 2019 Jan 17;11(1):163. doi: 10.3390/polym11010163 (PMC6401893; doi:10.3390/polym11010163)
Supplement: Supplementary file 1 [file polymers-11-00163-s001.pdf]

Supporting Information

Micelle Structure Details and Stabilities of Cyclic Block Copolymer Amphiphile and Its Linear Analogues

Brian J. Ree,[1] Toshifumi Satoh,*[,2] and Takuya Yamamoto*[,2]

[1]Graduate School of Chemical Sciences and Engineering, Hokkaido University, Sapporo 060-8628, Japan

[2]Faculty of Engineering, Hokkaido University, Sapporo 060-8628, Japan

Synthesis

CH_2Cl_2 (99%, Kanto Chemical) was distilled from CaH_2 before use. CuBr (99%, Nacalai Tesque), 4,4'-dinonyl-2,2'-dipyridyl (dNbpy) (97%, Aldrich), allyltributyl stannane (97%, Aldrich), *n*-hexane (Godo Yozai), the second-generation Hoveyda–Grubbs catalyst (Aldrich), and activated alumina (300 mesh, Wako Chemical) were used as received. *n*-Butyl acrylate (BA: 99%, Nacalai Tesque) was distilled under reduced pressure.

Poly(ethylene oxide) macroinitiator having one 2-bromoisobutyryl terminal group (PEO-1: number-average molecular weight $\overline{M}_{n,NMR}$ = 6070) was prepared by the esterification of poly(ethylene oxide) (PEO: $\overline{M}_{n,NMR}$= 6000, Aldrich) with 2-bromoisobutyryl bromide (98%, Aldrich) according to the procedure previously reported [1-3]. In the similar manner, another PEO macroinitiator having two 2-bromoisobutyryl terminal groups (PEO-2: $\overline{M}_{n,NMR}$= 3000) was prepared by the esterification of PEO ($\overline{M}_{n,NMR}$= 3350, Aldrich) with 2-bromoisobutyryl bromide (98%, Aldrich).

An amphiphilic linear *diblock* copolymer, poly(*n*-butyl acrylate)-*b*-poly(ethylene oxide) (PBA-*b*-PEO) was synthesized through atom transfer radical polymerization (ATRP) and subsequent Keck allylation, according to the method reported previously.[1] PEO-1 macroinitiator ($\overline{M}_{n,NMR}$ = 6070, 0.90 g, 0.15 mmol), BA (1.73 mL, 12.1 mmol), dNbpy (180.2 mg, 0.441 mmol), and CuBr (20.9 mg, 0.146 mmol) were put in a test tube. The mixture was degassed by three freeze–pump–thaw cycles, and the test tube was sealed under vacuum. The resulting suspension

was stirred at 90 °C for 3 h, cooled with liquid nitrogen, and allowed to warm to 20 °C. Allyltributylstannane (0.44 mL, 1.4 mmol) and toluene (2.9 mL) were added to the reaction mixture, and the resulting suspension was degassed by three freeze–pump–thaw cycles. The test tube was sealed under vacuum, and the suspension was stirred at 110 °C for another 18 h. The reaction mixture was precipitated in *n*-hexane, and the precipitate was dissolved in THF and filtered through a plug of alumina. The eluent was concentrated and precipitated in *n*-hexane and an aqueous NaCl solution to allow isolation of PBA-*b*-PEO as waxy solid (240.2 mg). The target polymer product was characterized using a nuclear magnetic resonance (NMR) spectrometer (JEOL JNM-ECS400) with proton (^{1}H) probe. Deuterated chloroform ($CDCl_3$) was used as a solvent, and the chemical shifts were reported relative to the signal of tetramethylsilane. ^{1}H NMR ($CDCl_3$, δ (ppm)): 0.94 (t, -$CO_2CH_2CH_2CH_2C\mathit{H}_3$), 1.15 (m, 6H, -$C(C\mathit{H}_3)_2$-), 1.18-2.30 (m, -$C\mathit{H}_2CH(CO_2$*n*-Bu)-, -$CH_2C\mathit{H}(CO_2$*n*-Bu)-, -$CO_2CH_2C\mathit{H}_2C\mathit{H}_2CH_3$, -$C\mathit{H}_2CH{=}CH_2$), 3.64 (m, -$OC\mathit{H}_2C\mathit{H}_2O$-, -$OCH_3$), 4.05 (m, -$CO_2C\mathit{H}_2CH_2CH_2CH_3$), 4.18 (m, 2H, -$C\mathit{H}_2OCOC(CH_3)_2$-), 5.01 (m, 2H, -$CH_2CH{=}C\mathit{H}_2$), 5.68 (m, 1H, -$CH_2C\mathit{H}{=}CH_2$).

From the ^{1}H NMR spectrum, the number of repeat unit in the PBA block was determined from the integrations of the BA unit' methyl peak (-$CO_2CH_2CH_2CH_2C\mathit{H}_3$) and the linker unit' dimethyl peak (-$C(C\mathit{H}_3)_2$-), while the number of repeat unit in the PEO block was estimated from the integrations of the EO unit' methylene peak (-$OC\mathit{H}_2C\mathit{H}_2O$-) and the linker unit' dimethyl peak (-$C(C\mathit{H}_3)_2$-). This analysis gave the composition and molecular weight of the linear block copolymer product: $[CH_3O(EO)_{126.2}CO]–[C(CH_3)_2(BA)_{8.8}CH_2CH{=}CH_2]$ and $\bar{M}_{n,NMR}$ = [5620]–[1210]. The polymer product was further analyzed using a gel permeation chromatography (GPC) system (Shodex GPC-101), which was equipped with a refractive index detector (Shodex RI-71S) and two columns (Shodex GPC KF-804L). The GPC system was calibrated with polystyrene standards. Tetrahydrofuran (THF) was used as an eluent at a flow rate of 1.0 mL/min at 40 °C. The linear diblock copolymer product was determined to have a number-average molecular weight $\bar{M}_{n,GPC}$ of 5950 and a polydispersity index (PDI) of 1.07.

An amphiphilic linear *triblock* copolymer, poly(*n*-butyl acrylate)-*b*-poly(ethylene oxide)-*b*-poly(*n*-butyl acrylate) having two allyl end groups (PBA-*b*-PEO-*b*-PBA) was synthesized through ATRP and subsequent Keck allylation, according to the method described above. PEO-2 macroinitiator ($\bar{M}_{n,NMR}$= 3000, 1.0 g, 0.31 mmol), BA (5.0 g, 39 mmol), dNbpy (760 mg, 1.9 mmol), and CuBr (90 mg, 0.63 mmol) were put in a test tube. The mixture was degassed by three freeze–pump–thaw cycles, and the test tube was sealed under vacuum. The resulting suspension was stirred

at 80 °C for 30 min, cooled with liquid nitrogen, and allowed to warm to 20 °C. Allyltributylstannane (0.96 mL, 3.1 mmol) and toluene (5.0 mL) were added to the reaction mixture, and the resulting suspension was degassed by three freeze–pump–thaw cycles. The test tube was sealed under vacuum, and the suspension was stirred at 110 °C for another 18 h. The reaction mixture was precipitated in *n*-hexane, and the precipitate was dissolved in THF and filtered through a plug of alumina. The eluent was concentrated and precipitated in *n*-hexane to allow isolation of the target product as colorless waxy solid (672 mg). The target polymer product was characterized by NMR spectroscopy. ^{1}H NMR ($CDCl_3$, δ (ppm)): 0.91 (t, -$CO_2CH_2CH_2CH_2CH_3$), 1.12 (m, 12H, -$C(CH_3)_2$-), 1.25-2.10 (m, -$CH_2CH(CO_2$*n*-Bu)-, -$CO_2CH_2CH_2CH_2CH_3$), 2.29 (m, -$CH_2CH(CO_2$*n*-Bu)-), 3.62 (s, -OCH_2CH_2O-), 4.03 (m, -$CO_2CH_2CH_2CH_2CH_3$), 4.15 (m, 4H, -$CH_2OCOC(CH_3)_2$-), 5.01 (m, 4H, -$CH_2CH{=}CH_2$), 5.66 (m, 2H, -$CH_2CH{=}CH_2$). The product was further determined: [$H_2C{=}CHCH_2(BA)_{5.1}C(CH_3)_2$]–[$COO(EO)_{69.1}CO$]–[$C(CH_3)_2(BA)_{5.1}CH_2CH{=}CH_2$], $\bar{M}_{n,NMR}$ = [740]–[3110]–[740], $\bar{M}_{n,GPC}$= 5850, and PDI = 1.11.

An amphiphilic *cyclic* diblock copolymer, poly(*n*-butyl acrylate)-*b*-poly(ethylene oxide) (cyclic PBA-*b*-PEO) was prepared from some of the above linear triblock block copolymer product; here, the linear PBA-*b*-PEO-*b*-PBA having two allyl end groups was used as the precursor. The second-generation Hoveyda–Grubbs catalyst (87 mg, 0.14 mmol) was added to a toluene solution (0.80 L) of PBA-*b*-PEO-*b*-PBA (200 mg, 44 μmol), and the resulting solution was stirred at 60 °C for 22 h. Ethyl vinyl ether (40 mL) was added to the reaction mixture to quench the catalyst. The color of the solution immediately changed from brown–green to brown. The mixture was further stirred at room temperature for 14 h. The reaction mixture was concentrated and subjected to silica gel column chromatography. The byproducts and impurities were first eluted with EtOAc, and the product was collected with MeOH. The crude product was fractionated by preparative GPC with $CHCl_3$ as an eluent at a flow rate of 3.5 mL/min to allow isolation of the target cyclic product (177 mg) in 88% yield. ^{1}H NMR ($CDCl_3$, δ (ppm)): 0.91 (t, -$CO_2CH_2CH_2CH_2CH_3$), 1.12 (m, 12H, -$C(CH_3)_2$-), 1.21-2.10 (m, -$CH_2CH(CO_2$*n*-Bu)-, -$CO_2CH_2CH_2CH_2CH_3$), 2.29 (m, -$CH_2CH(CO_2$*n*-Bu)-), 3.62 (s, -OCH_2CH_2O-), 4.01 (m, -$CO_2CH_2CH_2CH_2CH_3$), 4.14 (m, 4H, -$CH_2OCOC(CH_3)_2$-), 5.28 (m, 2H, cis and trans -$CH{=}CH$-). The NMR spectroscopy and GPC analyses gave the composition, molecular weight and polydispersity: [$C(CH_3)_2(BA)_{5.3}H_2CCH{=}CHCH_2(BA)_{5.3}$-$(CH_3)_2$]–[$COO(EO)_{67.7}CO$], $\bar{M}_{n,NMR}$ = [1500]–[3050], $\bar{M}_{n,GPC}$ = 4700, and PDI = 1.18.

X-ray scattering data analyses

Figure S3 shows representatives of the X-ray scattering intensity $I(q)$ data measured for the micelles of *l*-PBA-*b*-PEO, *l*-PBA-*b*-PEO-*b*-PBA, and *c*-PBA-*b*-PEO in aqueous solutions.

In general, the scattering intensity $I(q)$ of particles in a solvent medium is expressed by the following equation:[2-4]

$$I(q) = K_x N_p P(q) \cdot S(q) \qquad (1)$$

where K_x is a constant, N_p is the number of particles, $P(q)$ is the form factor of the particle, and $S(q)$ is the structure factor for the particles. q is the magnitude of the scattering vector defined as $q = (4\pi/\lambda)\sin\theta$ in which 2θ is the scattering angle and λ is the wavelength of the X-ray beam used. The $P(q)$ term contains the information of the shape and the size of the particle while the $S(q)$ term describes the interparticle distance as the following:[2,3]

$$S(q) = 1 + \frac{1}{N}\left\langle \sum_{l=1}^{N} \sum_{l' \neq 1}^{N} e^{-jq(r_l - r_{l'})} \right\rangle \qquad (2)$$

where r_l is the position of particle l. The first term of the structure factor equals 1 due to the perfect positional correlation stemming from the particle l itself. The second term is the interference function between particles.

For general solution scattering cases in dilute conditions, the interparticle distance $(r_l - r_{l'})$ become random and the interference function reaches 0,

$$\frac{1}{N}\left\langle \sum_{l=1}^{N} \sum_{l' \neq 1}^{N} e^{-jq(r_l - r_{l'})} \right\rangle \rightarrow 0\,. \qquad (3)$$

Then,

$$S(q) \approx 1\,, \qquad (4)$$

thereby allowing the approximation of the $S(q)$ term as unity over the entire q range. This approximation allows the isolation of $P(q)$ from the overall scattering intensity $I(q)$. Therefore, equation 1 can be rewritten as:

$$I(q) \cong K_x N_p P(q). \tag{5}$$

Dilute conditions, however, present the problem of having considerably low intensity in the high q region as opposed to the low q region, which is detected in much higher intensity. This problem causes the intensity in the high q region to be obscured by the background noise, producing significant errors in analysis of the scattering data. As a way of overcoming such low intensity, semidilute conditions are often used with the assumption that the interparticle interaction is low enough where $S(q) \approx 1$ over the entire q range. In this study, the X-ray scattering measurements were conducted with polymeric micelle solutions with concentration ranging from 1.0 to 5.0 mg/mL to obtain high quality scattering data with negligible $S(q)$.

Guinier analysis. The Guinier analysis is a model independent method based on the law of Guinier developed by André Guinier, which is expressed as the following equation:[2-4]

$$\ln I(q) = \ln I_o(q) - \frac{q^2 R_{g,G}{}^2}{3} \tag{6}$$

where $I_o(q)$ is the incident beam intensity. According to the law of Guinier, the radius of gyration $R_{g,G}$ of particles in solution can be approximated from the low q region of the scattering data. This approximation, however, must satisfy the two boundaries conditions: the particles must be globular and the maximum $qR_{g,G}$ must be less than 1.33. Within these boundary conditions, the obtained scattering data can be plotted into a *log* $I(q)$ *vs.* q^2 curve, in which the resulting slope of the curve m is used to yield the approximate $R_{g,G}$ of particles (i.e., micelles in this study) by the following expression:

$$R_{g,G} = \sqrt{-3m}. \tag{7}$$

Indirect Fourier transformation analysis. The indirect Fourier transformation (IFT) analysis is another model independent method that could provide more structural information than the Guinier analysis. Parameters such as radius of gyration R_g can be obtained through the IFT method,[4,5] which

transforms the scattering data to its real space analogue, the pair distance distribution function $p(r)$. The pair distance distribution function describes the probability of finding two scatterers separated by a distance r inside the particle (i.e., micelle). This data transformation bypasses the utilization of a specific parameterized model and directly derives the structural information from the scattering data. The scattering intensity can be expressed by the Fourier transformation of $p(r)$:[4,5]

$$I(q) = 4\pi \int_0^\infty p(r) \frac{\sin(qr)}{qr} dr \tag{8}$$

in which an estimate of $R_{g,IFT}$ can be derived based on $p(r)$ as follows:

$$R_{g,IFT} = \frac{\int_0^\infty p(r) r^2 dr}{2\int_0^\infty p(r) dr}. \tag{9}$$

In addition to the radius of gyration, the IFT method can offer the radial electron density distribution function $\rho(r)$, which is related to the pair distance distribution function in the following:[4,5]

$$p(r) = r^2 \int_{-\infty}^{\infty} \Delta\rho(x)\Delta\rho(x-r) dx \tag{10}$$

where r is the distance from the center of the micelle.

Spherical core-fuzzy shell model. The spherical core-fuzzy shell (CFS) model is based on a spherical particle composed of two phases, namely a dense core and a fuzzy shell.[4,6] For the spherical CFS particle, the $P(q)$ term in equation 5 can be expressed as a sum of two terms:[4,6]

$$P(q) = P_{shape}(q) + 4\pi\alpha \int_0^\xi r^2 \gamma(r) \cdot \frac{\sin(qr)}{qr} dr \tag{11}$$

where the first term, $P_{shape}(q)$, is the form factor of the overall spherical particle composed of a dense core and a fuzzy shell, and the second term (i.e., $P_{\text{blob}}(q)$) is the contribution from the density fluctuations that occur within the fuzzy shell. Here, the second term describes the Fourier transform of the correlation function $\gamma(r)$ of density fluctuations on length scales (r) smaller than the blob

radius, α is the amplitude of the blob scattering contribution, and ξ is the average correlation length. $\gamma(r)$ is equal to zero for $r > \xi$, but not equal to zero for $r \leq \xi$. For $r \leq \xi$, $\gamma(r)$ can be expressed by

$$\gamma(r) \propto r^{\mu-2} \tag{12}$$

where

$$\mu = \chi^{-1} - 1 \tag{13}$$

where χ is the Flory-Huggins parameter, which equals 3/5 for the good solvent condition, 1/2 for the Θ solvent condition, and 2/3 in the case that the molecules are stretched.[4,6]

In light of fuzzy-sphere,[6] $P_{shape}(q)$ for the core-fuzzy shell can be replaced by the following equation:

$$P_{CFS}(q, r_{c,CFS}, r_{CFS}, \sigma_{f,CFS}) = [A_{CFS}(q, r_{c,CFS}, r_{CFS}, \sigma_{f,CFS})]^2 \tag{14}$$

where

$$A_{CFS}(q, r_{c,CFS}, r_{CFS}, \sigma_{f,CFS}) = A_{CFS}(q, r_{c,CFS}, r_{CFS}) e^{-\frac{q^2 \sigma_{f,CFS}^2}{4}} \tag{15}$$

where A_{CFS} is the scattering amplitude of the core-shell structure with fuzziness, $r_{c,CFS}$ is the radius of the dense spherical core, r_{CFS} is the outer radius of core-fuzzy shell (i.e., total radius of the micelle), which is related to the shell thickness by $t_{f,CFS} = r_{CFS} - r_{c,CFS}$. $2\sigma_{f,CFS}$ is a measure of the width of the soft region in the shell part, and A_{CFS} is the scattering amplitude of the core-shell structure with sharp interface and uniform density distribution in core and shell, respectively. Further, the fuzziness of the shell part is defined by the ratio of $\sigma_{f,CFS}$ and $t_{f,CFS}$, because only shell region is fuzzy. The scattering amplitude of the core-shell is given by the following equation:[7]

$$A_{CFS}(q, r_{c,CFS}, r_{CFS}) = \Delta\rho_{core}[\Delta\rho_{shell} V_{shell} A(q, r_{CFS}) - (\Delta\rho_{shell} - \Delta\rho_{core}) V_{core} A(q, r_{c,CFS})] \tag{16}$$

where $A(q,r_{\mathrm{CFS}})$ and $A(q,r_{\mathrm{c,CFS}})$ can be calculated by the following equation:

$$P_{core}(q,r_{c,CFS}) = [A(q,r_{c,CFS})]^2 = \left[\frac{3[\sin(qr_{c,CFS}) - qr_{c,CFS}\cos(qr_{c,CFS})]}{(qr_{c,CFS})^2} \right]^2 \quad (17)$$

where P_{core} is the self-correlation term of the core, $A(q,r_{c,SCM})$ is the scattering amplitude of spherical core, and $r_{c,SCM}$ is the radius of core.

Two phase ellipsoid model. In this study, we have developed a new model dependent method as follows. This proposed structural model is ellipsoidal in shape and is composed of two phases: (i) a dense core and (ii) a corona. For this two layer ellipsoid particle, the $P(q)$ in equation 5 can be expressed by the sum of two terms in the following equation:

$$P(q) = P_{shape}(q) + P_{blob}(q)\,. \quad (18)$$

$P_{shape}(q)$ describes an ellipsoidal particle consisting of a core and a corona. The second term $P_{blob}(q)$ describes the density fluctuations smaller than the blob radius within the corona.

In $P_{shape}(q)$, the form factor describes a spheroid shape with a pair of equal semi-axes (R, R) and a distinct third semi-axis (εR) (i.e., an ellipsoid of gyration). The ellipsoidicity ratio ε in this model is defined as the ratio between the polar axis (R_p) and the two equatorial axes (R_e):

$$\varepsilon = \frac{R_p}{R_e}. \quad (19)$$

For a single spheroid with the equatorial radius of R (i.e., R_e), the form factor is described as:[8,9]

$$P_{shape}(q) = \int_0^{\frac{\pi}{2}} F^2[q,r(R_e,\varepsilon,\varphi)]\sin\varphi \; d\varphi \quad (20)$$

where $F[q,r(R_e,\varepsilon,\varphi)]$ is the scattering amplitude and φ is the modular angle of spheroid. The scattering amplitude is given as a function of the scattering vector q, and $r(R_e,\varepsilon,\varphi)$:

$$F[q,r(R_e,\varepsilon,\varphi)] = \frac{3\{\sin[qr(R_e,\varepsilon,\varphi)] - qr(R_e,\varepsilon,\varphi)\cos[qr(R_e,\varepsilon,\varphi)]\}}{[qr(R_e,\varepsilon,\varphi)]^3} \tag{21}$$

where

$$r(R_e,\varepsilon,\varphi) = R_e(\sin^2\varphi + \varepsilon^2\cos^2\varphi)^{\frac{1}{2}}. \tag{22}$$

In the case of two layer ellipsoid model, the overall form factor can be expressed as thusly:

$$P_{shape}(q) = \int_0^{\frac{\pi}{2}} [(\rho_{corona} V_{s,corona} F_{corona}^2) + \{(\rho_{corona} - \rho_{core}) V_{core} F_{core}^2\}] \sin\varphi \, d\varphi. \tag{23}$$

Additionally, each scattering amplitude term include an interface term that functions to describe the gradual decay of polymeric micelle's density along the radial direction. This is especially prevalent in the corona due to the solvation but, because polymeric micelles are soft particles, the core-corona interface also possesses a sizeable region with decaying density. As a result, the regions of fuzzy, nonideal interface could be described with scattering as the following equation:

$$F[q,r(R_e,\varepsilon,\varphi),t_f] = F[q,r(R_e,\varepsilon,\varphi)] \cdot e^{-\frac{q^2 t_f^2}{4}} \tag{24}$$

where $2t_f$ is the width (i.e., thickness) of the fuzzy interface in the core-corona interface and the corona-solvent interface.

The second term of the overall form factor, $P_{blob}(q)$, is expressed as the following:[6]

$$P_{blob}(q) = 4\pi\alpha \int_0^{\xi} r^2 \gamma(r) \frac{\sin(qr)}{qr} dr \tag{27}$$

which is same with the second term in equation 11. Again, for $r \leq \xi$, $\gamma(r)$ can be defined by equation 12; the μ in $\gamma(r)$ is defined by equation 13.

Three phase ellipsoid model. The final iteration of model dependent method is based on the three phase ellipsoid model in the literature.[10] This structural model is also ellipsoidal in shape and is composed of three phases: (i) a dense core, (ii) a dense corona, and (iii) a solvated corona. For this three layer ellipsoid particle, the $P(q)$ in equation 5 could expressed as identical as equation 18. In this case, however, the first term $P_{shape}(q)$ describes an ellipsoidal particle consisting of three layer phases, namely a core, a dense corona, and a solvated corona. The core is unpenetrated by solvent molecules and the corona is divided into two regions of different densities depending on the level of solvent penetration. The second term $P_{blob}(q)$ describes the density fluctuations smaller than the blob radius within the corona regions.

In $P_{shape}(q)$, the form factor takes a spheroid shape with a pair of equal semi-axes (R, R) and a distinct third semi-axis (εR). The ellipsoidicity ratio ε (the ratio between the polar axis (R_p) and the two equatorial axes (R_e)) in this model is defined in equation 19. The mathematical description of a spheroid with the equatorial radius of R (i.e., R_e) is defined in equations 20–22.

In the case of three layer ellipsoid model, the overall form factor can be expressed as thusly:

$$P_{shape}(q) = \int_0^{\frac{\pi}{2}} [(\rho_{s.corona} V_{s.corona} F_{s.corona}^2) + \{(\rho_{s.corona} - \rho_{d.corona}) V_{d.corona} F_{d.corona}^2\} + \{(\rho_{d.corona} - \rho_{core}) V_{core} F_{core}^2\}] \sin\varphi \, d\varphi \tag{28}$$

Additionally, each scattering amplitude term include an interface term that functions to describe the gradual decay of polymeric micelle's density along the radial direction. The soft material characteristic of polymer micelles give rise to a fuzzy, nonideal interface rather than a sharp interface between layers. This is in effect at the core-dense corona interface as well as at the dense corona-solvated corona interface and the solvated corona-solvent interface. As a result, the scattering amplitude term for each phase can be rewritten in the same manner as equation 24 where $2t_f$ is the width (i.e., thickness) of the corresponding fuzzy interface.

Moreover, the core radius, dense corona thickness, and solvated corona thickness within $P_{shape}(q)$ are assumed to possess Gaussian distribution $n(A)$:

$$n(A)=\frac{1}{\sqrt{2\pi}\sigma_A}\cdot e^{-\frac{(A-\overline{A})^2}{2\sigma_A^2}} \quad (29)$$

where A corresponds to the core radius or the dense corona thickness or the solvated corona thickness, $\overline{A}$ is the mean value of the structural parameter A, and σ_A is the standard deviation of A from $\overline{A}$. Therefore, equation 24 can be rewritten as:

$$F[q,r(R_e,\varepsilon,\varphi),t_f,\sigma_A]=n(A)F[q,r(R_e,\varepsilon,\varphi)]\cdot e^{-\frac{q^2t_f^2}{4}} \quad (30)$$

The second term of the overall form factor, $P_{blob}(q)$, is expressed in equation 27, where $r\leq\xi$, $\gamma(r)$ can be defined by equation 12; the μ in $\gamma(r)$ is defined by equation 13.

References

(1) Adachi, K.; Honda, S.; Hayashi, S.; Tezuka, Y. *Macromolecules* **2008**, *41*, 7898–7903.

(2) (a) Guinier, A.; Fournet, G. *Small Angle Scattering X-Ray*; Wiley, New York, 1955. (b) Glatter, O.; Kratky, O. *Small Angle X-ray Scattering*; Academic, New York, 1982. (c) Roe, R.-J. *Methods of X-Ray and Neutron Scattering in Polymer Science*; Oxford U. Press, New York, 2000.

(3) (a) Shin, S. R.; Jin, K. S.; Lee, C. K.; Kim, S. I.; Spinks, G. M.; So, I.; Jeon, J.-H.; Kang, T. M.; Mun, J. Y.; Han, S.-S.; Ree, M.; Kim, S. J. *Adv. Mater.* **2009**, *21*, 1907–1910. (b) Jin, S.; Higashihara, T.; Jin, K. S.; Yoon, J.; Heo, K.; Kim, J.; Kim, K.-W.; Hirao, A.; Ree, M. *J. Phys. Chem. B* **2010**, *114,* 6247–6257. (c) Jin, K. S.; Shin, S. R.; Ahn, B.; Heo, K.; Kim, S. J.; Ree, M. *J. Phys. Chem. B* **2009**, *113*, 1852–1856. (d) Jin, K. S.; Rho, Y.; Kim, J.; Kim, H.; Kim, I. J.; Ree, M. *J. Phys. Chem. B* **2008**, *112*, 15821–15827.

(4) Heo, K.; Kim, Y. Y.; Kitazawa, Y.; Kim, M.; Jin, K. S.; Yamamoto, T.; Ree, M. *ACS Macro Lett.* **2014**, *3*, 233−239.

(5) (a) Glatter, O. J. *Appl. Crystallogr.* **1977**, *10*, 415−421. (b) Glatter, O. *J. Appl. Crystallogr.* **1980**, *13*, 577–584.

(6) Rathgeber, S.; Monkenbusch, M.; Kreitschmann, M.; Urban, V.; Brulet, A. *J. Chem. Phys.* **2002**, *117*, 4047–4062.

(7) Hayter, J. B.; Penfold, J. *Coll. Polym. Sci.* **1983**, *261*, 1022–1030.

(8) Pedersen, J. S. *Adv. Colloid Interface Sci.* **1997**, *70*, 171–210.

(9) Pedersen, J. S.; Gerstenberg, M. C. *Macromolecules* **1996**, *29*, 1363–1365.

(10) Ree, B. J.; Satoh, Y.; Jin, K. S.; Isono, T.; Kim, W. J.; Kakuchi, T.; Satoh, T.; Ree, M. *NPG Asia Materials* **2017**, *9*, e453.

Table S1 Structural parameters of micelles of the amphiphilic block copolymers obtained from Guinier and indirect Fourier transform (IFT) analyses

Structural parameter	Amphiphilic block copolymer		
	l-PBA-*b*-PEO	*l*-PBA-*b*-PEO-*b*-PBA	*c*-PBA-*b*-PEO
Guinier analysis			
$R_{g,G}{}^{a}$ (nm)	7.58	5.55	4.84
$\lvert R_{g,G} - R_{g,IFT} \rvert$ (nm)	0.05	0.45	0.01
IFT analysis			
$R_{g,IFT}{}^{b}$ (nm)	7.63	5.10	4.85
$D_{max}{}^{c}$ (nm)	28.70	13.00	12.50
$R_{max}{}^{d}$ (nm)	8.32	6.72	6.44
$R_{max}/R_{g,IFT}$	1.09	1.32	1.33

[a]Radius of gyration of micelle determined via Guinier analysis. [b]Radius of gyration of micelle determined via IFT analysis. [c]Maximum dimension (i.e., diameter) of micelle determined from the *p*(*r*) function. [d]Radius of micelle determined from the peak maximum of the *p*(*r*) function.

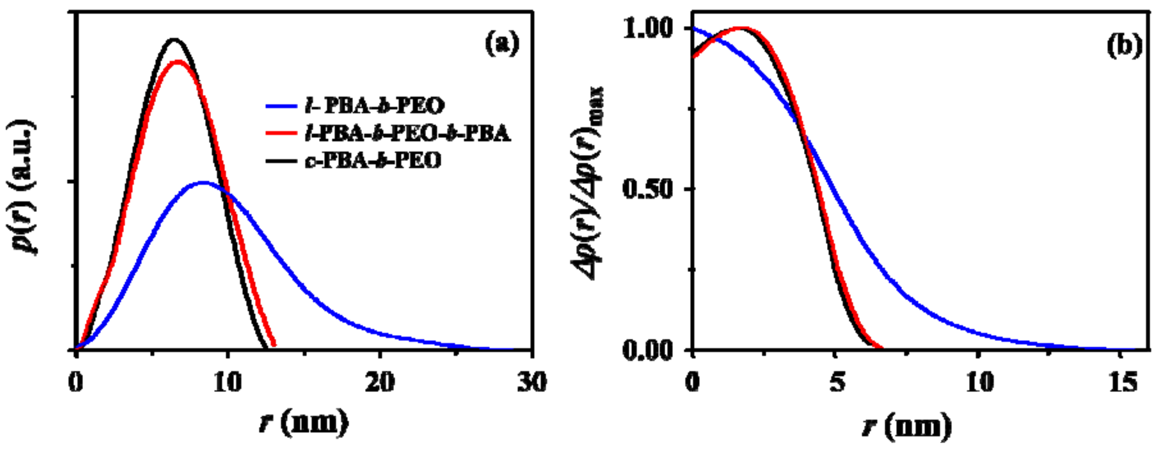

Figure S1. (a) Pair distance distribution functions *p*(*r*) and (b) normalized radial density distribution functions *ρ*(*r*) obtained by IFT analysis of the X-ray scattering data.

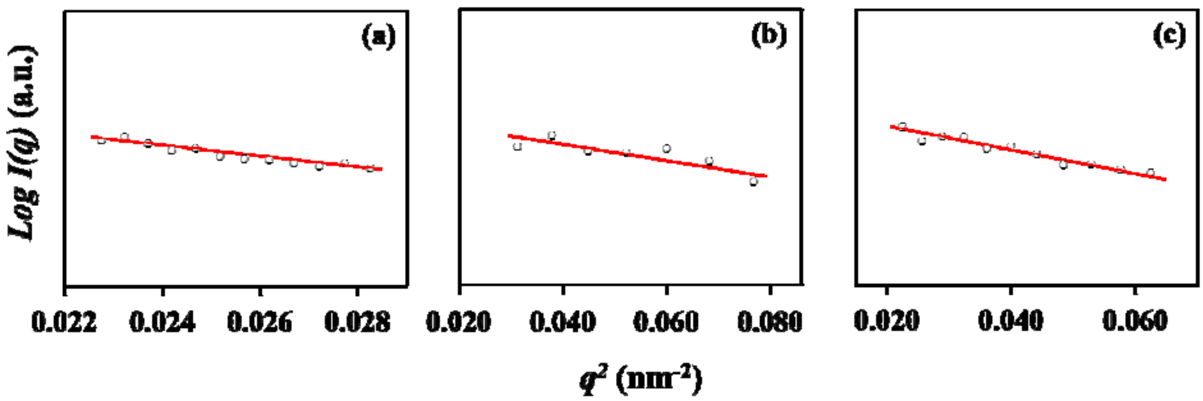

Figure S2. Guinier plots of the X-ray scattering data of micelles: (a) *l*-PBA-*b*-PEO; (b) *l*-PBA-*b*-PEO-*b*-PBA; (c) *c*-PBA-*b*-PEO. The white dot symbols are the measured data and the red solid lines represent the profiles obtained by fitting the data using the Guinier law.

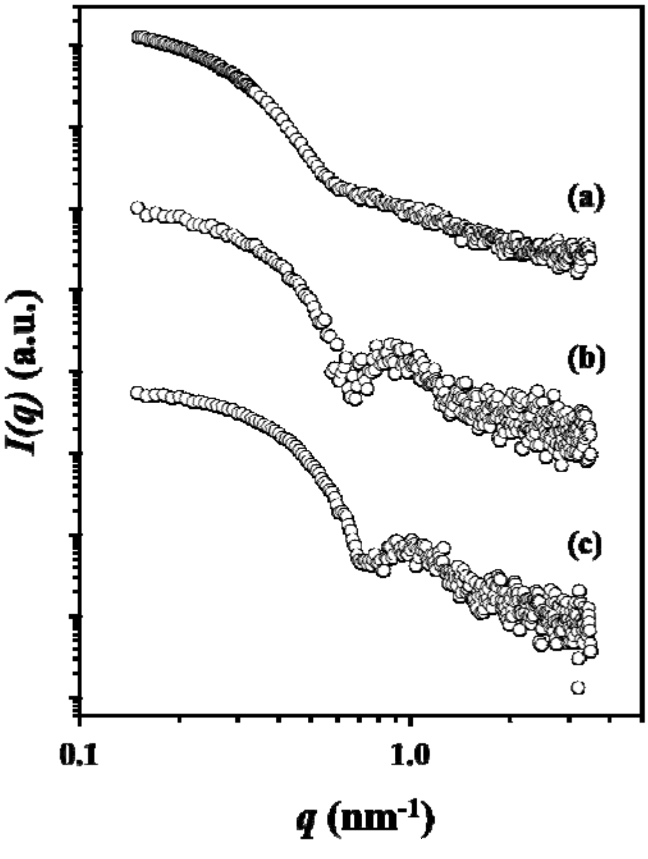

Figure S3. Representatives of the X-ray scattering intensity $I(q)$ profiles measured for the micelles of amphiphilic block copolymers formed in deionized water with a polymer concentration of 0.5 wt % at 25 °C: (a) *l*-PBA-*b*-PEO; (b) *l*-PBA-*b*-PEO-*b*-PBA; (c) *c*-PBA-*b*-PEO. $q = (4\pi/\lambda)\sin\theta$ in which 2θ is the scattering angle and λ is the wavelength of the X-ray beam used.
